# The Moroccan *Meska Horra*: A Natural Candidate for Food and Therapeutic Applications

**DOI:** 10.3390/foods14071158

**Published:** 2025-03-26

**Authors:** Abdessamad Beraich, Burak Dikici, Hammadi El Farissi, Daniela Batovska, Krastena Nikolova, Yousra Belbachir, Anass Choukoud, Nour Eddine Bentouhami, Abdeslam Asehraou, Abdelmoneam Talhaoui

**Affiliations:** 1 Laboratory of Environment and Applied Chemistry (LCAE), Team Physical Chemistry of the Natural Resources and Processes, Department of Chemistry, Faculty of Sciences, Mohamed First University, Oujda 60000, Morocco; h.elfarissi@uae.ac.ma (H.E.F.); yousra.belbachir@ump.ac.ma (Y.B.); anass.choukoud@ump.ac.ma (A.C.); a.talhaoui@ump.ac.ma (A.T.); 2 Department of Mechanical Engineering, Faculty of Engineering, Ataturk University, 25240 Erzurum, Turkey; burakdikici@gmail.com; 3 Chemical Engineering for Resources Valorization Group—UAE/L01FST, Faculty of Sciences and Technology, Abdelmalek Essaadi University, Tangier 90010, Morocco; 4 Institute of Chemical Engineering, Bulgarian Academy of Sciences, Acad. G. Bonchev Str., Bl. 103, 1113 Sofia, Bulgaria; danielabatovska@gmail.com; 5 Department of Physics and Biophysics, Faculty of Pharmacy, Medical University of Varna, 9000 Varna, Bulgaria; 6 Laboratory of Bioresources Biotechnology, Ethnopharmacology, and Health, Team Microbiology, Faculty of Sciences, Mohammed First University, Oujda 60000, Morocco; noureddine.bentouhami@ump.ac.ma (N.E.B.); a.asehraou@ump.ac.ma (A.A.)

**Keywords:** essential oil, head spaces extraction, hydrodistillation, *Pistacia lentiscus* L. resin, SPME, US-SPME, molecular docking

## Abstract

Mastic gum (*Pistacia lentiscus* L. resin), traditionally known as *Meska Horra* in Morocco, is valued for its bioactive properties, although its composition varies depending on its geographical origin. The essential oil profile is also influenced by the extraction method used. This study evaluates the chemical composition, bioactivity, and extraction efficiency of *Meska Horra* essential oil from eastern Morocco. Specifically, it explores its potential as a natural preservative and functional food ingredient by comparing various extraction methods and their impact on the profiles of volatile compounds. The essential oil obtained through hydrodistillation yielded 1.4% and met the standards of the European Pharmacopoeia despite differing in composition and quantity from Chios mastic gum. The major constituents were α-pinene, β-pinene, and D-limonene, comprising 55% of the oil. The oil demonstrated significant antioxidant and antimicrobial activity, supporting its potential application in food preservation. Molecular docking indicated that caryophyllene and its oxide are key bioactive compounds, although their effectiveness may be enhanced by synergistic interactions. Comparative analysis of extraction methods showed that headspace (HS) extraction captured highly volatile monoterpenes, while solid-phase microextraction (SPME) and ultrasound-assisted SPME (US-SPME) were more effective at extracting compounds such as *cis*-ocimene and limonene. US-SPME also extracted higher levels of *m*-cymene but lower levels of α-pinene. These findings highlights the importance of optimizing extraction methods and further investigating the role of synergistic effects in foods and pharmaceutical applications.

## 1. Introduction

Mastic gum is a natural resin derived from the *Pistacia lentiscus* L. tree, an evergreen species native to the Mediterranean region It is especially well known for being traditionally harvested on the Greek island of Chios, where its production has been practiced for centuries [1].

In the food industry, Chios mastic gum is widely used as a flavoring and texturizing agent in baked goods, confections, and dairy products. Its interaction with food components influences texture and consistency across various formulations. In fermented dairy products, it has been shown to support probiotic cultures, contributing to improved nutritional quality. Furthermore, its functional properties have been explored in food products aimed at promoting digestive health [2,3,4].

As a natural flavor enhancer, Chios mastic gum is valued for its distinctive aroma and is incorporated into a wide range of culinary applications. It is also a key ingredient in traditional beverages, where it enhances both flavor and overall sensory appeal. More recently, its potential as a coffee flavoring has drawn interest, further expanding its role in the food industry [2,3,5].

In Morocco, *P. lentiscus* L. grows in various regions, particularly in the northeast [6]. Its resin is widely used in traditional cuisine, where it enhances the flavor and texture of *chebakia*, a sesame cookie, and *sellou* (also known as *sfouf*), a sweet, nutrient-rich confection made from browned flour, toasted sesame seeds, and almonds. It is incorporated into *almond briouats*, crispy pastries filled with sweet almond paste, and occasionally in *pastilla*, a savory-sweet, layered pie. Beyond its culinary applications, *Meska Horra* is traditionally burned to enhance the sensory quality of drinking water and to scent water jars [7,8].

The flavoring properties of mastic gum are attributed to its essential oil, which comprises approximately 3% of the resin [3]. Its main constituents include terpenes such as α-pinene (63–77%), β-myrcene (8–25%), β-pinene (2–3%), β-caryophyllene (1–2%), and D-limonene (1–2%). These compounds have been associated with a variety of biological activities such as antioxidant [9], antimicrobial [9], anti-inflammatory, [10] anticancer [11], and antidiabetic effects [12].

Importantly, these terpenes contribute significantly to the bioactivity of the resin. The antibacterial and antifungal properties of mastic gum have been well documented. Several studies have shown that Chios mastic gum inhibits the growth of pathogenic bacteria such as *Staphylococcus aureus*, *Escherichia coli*, and *Pseudomonas aeruginosa* [5]. It has also demonstrated antifungal activity against yeasts like *Candida glabrata*, indicating its potential as a natural antifungal agent [6].

Clinical and preclinical research further supports the anti-inflammatory properties of mastic resin, which can suppress inflammatory responses by downregulating pro-inflammatory cytokines and inhibiting enzymes such as COX-2 [7]. These findings suggest its potential therapeutic application in managing chronic inflammatory conditions, including gastrointestinal disorders [8].

Moreover, mastic gum has shown promising antitumor activity in *in vitro* models of colorectal and lung carcinoma, further supporting its value as a multifunctional natural product [13,14]. The biological effects observed in these studies are closely linked to the chemical profile of the essential oil, emphasizing the pharmacological relevance of its terpene-rich composition.

The composition of the essential oil varies depending on the time of resin collection and the interval between its exudation from the trunk and its harvesting [2]. Most studies focus on the essential oil composition of *P. lentiscus* L. leaves and fruits, with limited research on the resin’s chemical variability across different environmental conditions. However, insights from leaf and fruit studies suggest that environmental factors such as geography, climate, soil composition, and altitude significantly influence the plant’s secondary metabolite profile. For instance, variations in essential oil composition have been observed in *P. lentiscus* L. leaves from different Mediterranean regions, attributed to environmental conditions [15]. Additionally, soil parameters and bioclimatic characteristics have been shown to affect the essential oil composition of *P. lentiscus* L. in Spain [16]. These findings highlight the need for further research to understand how these factors specifically impact the resin’s chemical composition.

In our study, the general composition of the essential oil of the Moroccan *P. lentiscus* L. resin was found to be broadly similar to that of Chios mastic gum. However, subtle variations in the relative concentrations of key volatile compounds suggest an influence of local environmental conditions.

To explore these aspects in depth, the present study focuses on the essential oil of *P. lentiscus* L. resin collected in eastern Morocco. A traditional extraction method—hydrodistillation—was employed to assess its effect on the yield and recovery of bioactive constituents.

The chemical composition of the essential oil was then analyzed using gas chromatography–mass spectrometry (GC-MS). In parallel, modern non-destructive techniques, including headspace (HS) analysis, solid-phase microextraction (SPME), and ultrasound-assisted SPME (US-SPME), were applied to obtain a comprehensive volatile profile. These techniques are widely recognized as non-destructive and effective for profiling volatile compounds [13,14,17].

This study aims to evaluate not only the chemical composition of the Moroccan resin but also its biological activities, with a particular focus on antioxidant, antibacterial, and antifungal properties. Additionally, a molecular docking approach was used to predict the interactions between major volatile compounds and microbial targets, providing insight into the possible mechanisms of action underlying the observed bioactivities.

By comparing different extraction techniques and analyzing the essential oil’s functional properties, this work contributes new data on an underexplored population of *P. lentiscus* L. and supports its potential application as a natural preservative or functional ingredient in the food industry. The findings also offer broader implications for the pharmaceutical and cosmetic sectors, where interest in plant-based bioactives continues to grow.

## 2. Materials and Methods

### 2.1. P. lentiscus L. Resin

**Figure 1:**
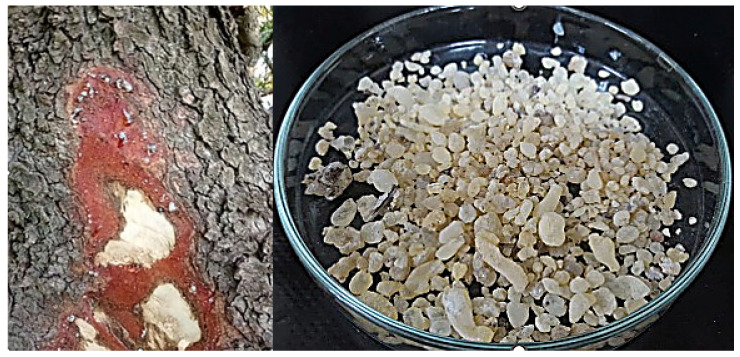
*Pistacia lentiscus* L. resin: (**Left**) Exudation from trunk incisions in the eastern region of Morocco; (**Right**) purified resin after collection and processing.

The *P. lentiscus* L. resin was collected from a forest located in the eastern region of Morocco, approximately 35–40 km from the Faculty of Science in Oujda, at an elevation of 1144 m (34°31′04″ N, 1°50′35″ W) (Figure 1). The collection followed traditional methods, involving the annual pruning of lower tree branches between mid-January and February. This practice promotes better air circulation and light penetration, which supports photosynthesis and aids in drying of the resin, ultimately contributing to a higher yield of by-products.

Resin collection was carried out between July and October by making incisions in the trunk, inducing the exudation of a highly viscous liquid suspension.

After collection, the raw resin was washed with water to remove dust and other surface impurities, and then dried in an oven at a carefully controlled temperature of 25–32 °C for seven days. This temperature range was chosen to minimize the loss or degradation of volatile compounds, based on literature indicating that drying below 40 °C generally preserves essential oil composition [18]. Although the referenced study focused on *P. lentiscus* L. leaves rather than resin, it showed that drying between 25 °C and 40 °C did not significantly alter the essential oil’s chemical profile—suggesting that similar conditions are suitable for resin to maintain the integrity of bioactive constituents.

To verify that no significant degradation occurred during drying, GC-MS analysis was conducted and the results were compared with literature values, confirming that the main volatile compounds—such as α-pinene, β-pinene, and D-limonene—were well preserved [19].

### 2.2. Hydrodistillation

Essential oil extraction was carried out using a Clevenger-type hydrodistillation apparatus (Sigma-Aldrich, Burlington, VT, USA). A total of 25 g of *P. lentiscus* L. resin was distilled with 500 mL of distilled water for three hours, or until no additional volatile compounds were released. The collected essential oil was carefully separated using a micropipette, transferred into a clean sample vial, and dried over anhydrous sodium sulfate. The oil was then stored at 4 °C until further analysis by GC-MS and for subsequent bioactivity assays.

### 2.3. Headspace Analysis (HS)

Headspace analysis was performed using a multifunctional EST Analytical FLEX Autosampler equipped with a 2.5MF-CTC-EGT-HS-5.6/0.63H 2.5 mL syringe (09W-416631A, Trajan Scientific, Ringwood, Australia). Approximately 1.5 g of finely powdered resin was placed into a 20 mL glass vial and sealed with a polytetrafluoroethylene (PTFE)-lined silicone rubber cap. The sample vial was then heated to 80 °C for 5 to 10 min under continuous mechanical agitation to facilitate the release of volatile compounds into the headspace. The fiber was subsequently exposed to the vapor phase to allow compound adsorption, followed by thermal desorption in the GC-MS injector at 250 °C for analysis.

### 2.4. Solid-Phase Microextraction (SPME)

SPME analysis was conducted using an EST Analytical FLEX Autosampler equipped with a 75 µm carboxen/polydimethylsiloxane (CAR/PDMS) fiber (Sigma-Aldrich, USA). A total of 1.5 g of powdered *P. lentiscus* resin was placed into a glass vial and sealed with a polytetrafluoroethylene (PTFE)-lined silicone rubber cap. The vial was heated to 60 °C and mechanically agitated for 20 min to facilitate the release of volatile compounds. Following exposure, the fiber was inserted into the GC-MS injector using a syringe and thermally desorbed at 250 °C for analysis.

### 2.5. Ultrasonic Solid-Phase Microextraction (US-SPME)

For US-SPME, 1.5 g of powdered resin was mixed with 6.5 mL of double-distilled water in a sealed glass vial. The vial was placed in an ultrasonic bath at 25 °C for 12 min to enhance the extraction of volatile constituents. Following sonication, the sample was incubated at 60 °C with mechanical agitation for 20 min. The fiber was then inserted into the GC-MS injector via a syringe and thermally desorbed at 250 °C for subsequent analysis.

### 2.6. GC/MS Analysis

The chemical composition of the essential oil, as well as the volatile compounds extracted via SPME and US-SPME, was analyzed using a Shimadzu GC-MS-QP2010 Ultra system (Shimadzu Corporation, Kyoto, Japan). The system was equipped with an Agilent DB-Wax high-polarity capillary column (polyethylene glycol stationary phase, 30 m × 0.25 mm × 0.25 µm). High-purity helium gas (99.9%) was used as the carrier, with a constant column flow rate of 1.33 mL/min. The GC was coupled to a QP2010 mass spectrometer for compound detection.

The oven temperature program was initiated at 40 °C and increased to 250 °C at a rate of 10 °C/min. The mass spectrometer operated in scan mode over a mass range of 40–400 *m*/*z*, with a scan speed of 1250 Hz and an ionization voltage of 70 eV. Each sample was injected in split mode with a split ratio of 1:10 and an injection volume of 1 µL.

Compound identification was performed by comparing retention times and mass spectral fragmentation patterns with those in the Wiley 9 and NIST 11 (W9N11) mass spectral libraries. Data acquisition and processing were carried out using LabSolutions software SC [20].

### 2.7. Free-Radical Scavenging Activity

#### 2.7.1. DPPH Assay

The DPPH free-radical scavenging activity of the essential oil was evaluated following the protocol described by Beraich et al., with slight modifications [21]. Briefly, 600 µL of essential oil at varying concentrations (1–100 mg/mL) was mixed with 2400 µL of a 0.1 mM DPPH solution prepared in ethanol. The mixture was incubated in the dark at room temperature for 60 min.

Absorbance was measured at 517 nm using a Thermo Scientific MULTISKAN GO Microplate Spectrophotometer (version 1.00.40, Thermo Fisher Scientific, Waltham, MA, USA). Ethanol served as the blank control. The percentage of DPPH radical inhibition was calculated using the following equation:

%Inhibition = [(A_a_ − A_b_)/A_a_] × 100,

where A_a_ is the absorbance of the negative control (DPPH solution without sample), and A_b_ is the absorbance of the test sample or positive control. The concentration of essential oil required to inhibit 50% of the DPPH radicals (IC_50_) was determined from the plotted dose–response curve.

#### 2.7.2. ABTS Assay

The ABTS radical scavenging activity was assessed following the method described by Ahmed et al., with slight modifications [22]. The ABTS•⁺ radical cation solution was prepared by mixing 9.5 mL of 0.7 M ABTS with 0.25 mL of 0.1 M potassium persulfate, followed by dilution to a final volume of 10 mL with distilled water. The mixture was incubated in the dark at room temperature for 18 h. Prior to use, the solution was diluted with 0.1 M potassium phosphate buffer (pH 7.4) to reach an absorbance of 0.70 ± 0.02 at 734 nm.

Essential oil samples were prepared in ethanol at concentrations ranging from 1 to 100 mg/mL. For each measurement, 600 µL of the sample was mixed with 2400 µL of the diluted ABTS solution in a test tube and vortexed thoroughly. Absorbance was measured at 734 nm using a Thermo Scientific MULTISKAN GO Microplate Spectrophotometer (version 1.00.40, Thermo Fisher Scientific, Waltham, MA, USA).

The percentage of ABTS radical inhibition was calculated using the following formula:

%Inhibition = [(A_c_ − A_d_)/A_c_] × 100,

where A_c_ is the absorbance of the control and A_d_ is the absorbance of the test sample or positive control. The IC_50_ value (the concentration required to achieve 50% inhibition) was calculated based on the dose–response curve, as previously described [21].

### 2.8. Evaluation of Antimicrobial Activity

The antimicrobial activity of the essential oil was evaluated against selected bacterial and fungal strains, obtained from the Microbial Biotechnology Laboratory, Faculty of Science, Oujda, Morocco. The tested microorganisms included:

- Gram-positive bacteria: *Staphylococcus aureus* (ATCC 6538) and *Micrococcus luteus* (LB 14110).
- Gram-negative bacteria: *Escherichia coli* (ATCC 10536) and *Pseudomonas aeruginosa* (ATCC 15442).
- Molds: *Aspergillus niger* and *Geotrichum candidum*.
- Yeasts: *Candida glabrata* and *Rhodotorula glutinis*.

#### 2.8.1. Culture Preparation

Bacterial strains were cultured on Mueller–Hinton agar (MHA) and incubated at 37 °C for 18 h prior to antimicrobial testing. The bacterial suspensions were standardized to 10^6^ CFU/mL by adjusting the optical density (OD) at 600 nm using an Epoch 2 Microplate Spectrophotometer (BioTek Instruments, Inc., based in Winooski, VT, USA).

Molds (*A. niger* and *G. candidum*) were grown on potato dextrose agar (PDA) at 25 °C for seven days. Spores were then harvested, suspended in sterile distilled water with 0.05% Tween 80, and standardized to 10^6^ spores/mL using a hemocytometer. Yeasts (*C. glabrata* and *R. glutinis*) were cultured on PDA at 28 °C for 48 h, and cell concentrations were adjusted to 10^6^ cells/mL based on absorbance at 600 nm.

#### 2.8.2. Disc Diffusion Assay

The antimicrobial activity of the essential oil was assessed using the disc diffusion method. Standardized microbial suspensions were evenly spread on MHA plates (for bacteria) and PDA plates (for fungi). Sterile filter paper discs (6 mm diameter, Whatman No. 3) were impregnated with 20 µL of the essential oil placed on the agar surface.

Gentamicin (1 mg/mL) was used as a positive control for bacterial strains, while cycloheximide (1 mg/mL) served as the positive control for fungal strains. Plates were incubated at 37 °C for bacterial cultures and at (25–28) °C for fungal cultures. The diameter of the inhibition zones was measured after incubation. All assays were performed in triplicate.

#### 2.8.3. Minimum Inhibitory Concentration (MIC) Determination

The minimum inhibitory concentration (MIC) was determined using the broth microdilution method in 96-well microplates, following the protocol described by [23].

For bacterial strains, the essential oil was serially diluted in Mueller–Hinton broth (MHB) supplemented with 0.15% agar, across a concentration range of 8% to 0.0015%. The bacterial inoculum was standardized to 10^6^ CFU/mL, and the plates were incubated at 37 °C for 24 h.

For fungal strains, serial dilutions of the essential oil were prepared in potato dextrose broth (PDB) supplemented with 0.15% agar, following the same concentration range. Fungal suspensions were standardized to 10^6^ cells/mL for yeasts and 10^6^ spores/mL for molds Microplates were incubated at 28 °C for 48 h.

After incubation, 20 µL of resazurin solution was added to each well to assess microbial viability. A color change indicated microbial growth, allowing for MIC determination based on the lowest concentration with no visible color shift.

### 2.9. Molecular Docking Studies

Molecular docking was performed using AutoDock Vina (v1.1.2) and AutoDockTools (v1.5.7) to evaluate the interactions between bioactive compounds and selected target proteins. These simulations aimed to provide insights into the potential antioxidant, antibacterial, and antifungal mechanisms of action of the compounds identified in the essential oil.

#### 2.9.1. Protein Selection and Preparation

Target proteins were chosen based on their biological roles in microbial survival and oxidative stress responses. The following protein structures were retrieved from the RCSB Protein Data Bank (PDB):

- Posaconazole (PDB ID: 5FSA)—A fungal cytochrome P450 inhibitor.
- Adenosine-5′-triphosphate synthase (PDB ID: 2ZDQ)—Involved in bacterial energy metabolism.
- Novobiocin resistance protein (PDB ID: 4URN)—A bacterial DNA gyrase inhibitor target.
- Adenosine-5′-diphosphate ribose hydrolase (PDB ID: 2CDU)—Associated with bacterial stress response.
- Pterin-6-Yl-methyl-monophosphate binding protein (PDB ID: 2VEG)—A bacterial enzyme cofactor.

The protein structures were prepared by removing water molecules and non-essential ligands using AutoDock Tools v1.5.7. Polar hydrogens were added, and Gasteiger charges were assigned to optimize docking accuracy.

#### 2.9.2. Ligand Preparation and Docking Procedure

The selected bioactive compounds were retrieved from the PubChem database in SDF format and converted to PDBQT format using Open Babel 3.1.0 software. Energy minimization was performed using AutoDock 4.2 Tools.

Molecular docking simulations were conducted using AutoDock Vina v1.5.7, with a grid box defined around the active site of each target protein. The Lamarckian genetic algorithm was employed for flexible docking, generating multiple conformations per ligand. Binding affinities were ranked based on the lowest binding energy (kcal/mol), with lower scores indicating stronger ligand–protein interactions.

#### 2.9.3. Analysis of Docking Results

Docking scores and molecular interactions—including hydrogen bonding, hydrophobic interactions, and van der Waals forces—were examined to evaluate the binding efficiency and stability of each ligand–protein complex. The docking poses and interaction details were visualized and analyzed using Discovery Studio Visualizer and PyMOL for further structural interpretation.

### 2.10. Statistical Analysis

Statistical analysis was performed using SPSS 23.0 software (SPSS Inc., Chicago, IL, USA). Duncan’s multiple range test was applied to identify statistically significant differences between the extracts with respect to their chemical composition. A significance threshold of *p* < 0.05 was used.

Descriptive statistics were employed for molecular docking results. Box plot visualizations of binding energies were also generated using SPSS to illustrate the distribution and variability of docking scores.

## 3. Results

### 3.1. Extraction Yield from Hydrodistillation

The essential oil of *P. lentiscus* L. resin was extracted via hydrodistillation, yielding 14 mL per kilogram of resin (1.4% yield), with a measured density of 0.8646 g/cm³. The obtained oil had a pale-yellow appearance and a strong, persistent aroma, indicative of its richness in volatile and bioactive constituents.

### 3.2. GC/MS Analysis of P. lentiscus L. Resin Essential Oil and Extracts

#### 3.2.1. Chemical Composition of *P. lentiscus* L. Resin Essential Oil

GC-MS analysis of the hydrodistilled essential oil revealed 40 compounds. Monoterpenoids dominated the composition, accounting for 96% of the total detected volatiles, with oxygenated monoterpenes contributing approximately 27% of this fraction (Table 1). The major constituents were monoterpene hydrocarbons, including α-pinene (26%), β-pinene (19%), D-limonene (10%), *E*,*E*-2,6-dimethyl-3,5,7-octatriene-2-ol (4%), *p*-cymene (4%), myrcenol (4%), myrcene (3%), camphene (3%), α-campholenal (3%), and *p*-mentha-1,5-dien-8-ol (3%).

The only non-terpenoid volatile detected was 2-ethyl-4,5-dimethylphenol (1%), a compound commonly found in rosemary essential oil, which may contribute to the unique aroma profile and bioactivity of the *P. lentiscus* L. resin oil.

#### 3.2.2. Chemical Composition of *P. lentiscus* L. Resin Extracts

The chemical composition of the resin extracts varied according to the extraction method applied (Table 1). US-SPME produced the extract with the highest proportion of monoterpenoids (91%), followed by SPME at 89%, and HS at 71%. The dominant components identified were as follows:

- US-SPME: *cis*-Ocimene (46%), *m*-cymene (10%), D-limonene (6%), verbenone isomer (4%), α-pinene (4%), and verbenene (3%).
- SPME: *cis*-Ocimene (54%), D-limonene (6%), β-pinene (5%), camphene (3%), and (*Z*)-hexadec-7-enal (3%).
- HS: α-Pinene (14%), camphene (12%), β-pinene isomer (10%), tridec-3-ene (9%), 3-carene (7%), *p*-mentha-1(7),8(10)-dien-9-ol (7%), tricyclene (6%), *trans*-dodec-2-en-1-al (5%), *E*-dec-2-enal (4%), and sabinene (3%).

Among the three techniques, HS extraction yielded the highest proportion of non-terpenoid volatiles (28%), which primarily long-chain alcohols and aldehydes. These findings highlight the significant influence of the extraction method on the qualitative and quantitative composition of volatile constituents in *P. lentiscus* L. resin.

The standard deviation of the means is not presented in the table, as the standard error of the method was determined to be less than 3%. This low error margin indicates high measurement precision and minimal variation between replicates, rendering the standard deviation statistically insignificant for data interpretation.

### 3.3. Antioxidant Activity of P. lentiscus L. Resin Essential Oil

The antioxidant activity of *P. lentiscus* L. resin essential oil was assessed using DPPH and ABTS assays, with ascorbic acid serving as the positive control. Ascorbic acid exhibited an IC_50_ value of 15 ± 2 μg/mL.

**Figure 2:**
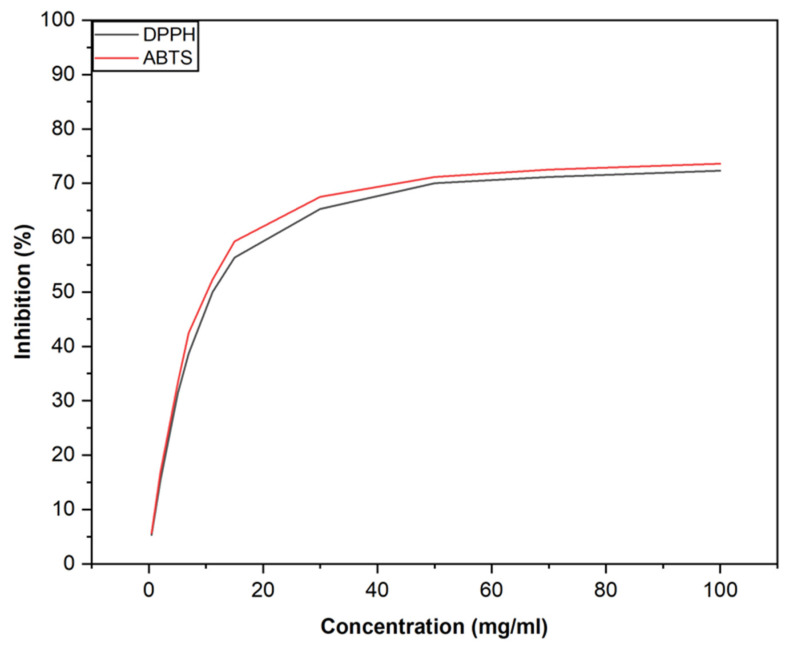
Antioxidant activity of *Pistacia lentiscus* L. resin essential oil evaluated by DPPH and ABTS assays.

In the DPPH assay, the essential oil showed an IC_50_ value of 11.1 ± 0.1 mg/mL. In the ABTS assay, the IC_50_ values were 10.9 ± 0.1 mg/mL for the essential oil and 14.1 ± 2 μg/mL for the positive control. These results indicate that the essential oil possesses moderate antioxidant activity, as illustrated in Figure 2.

### 3.4. Antimicrobial Properties of P. lentiscus L. Resin Essential Oil

In the agar diffusion assay, the essential oil of *P. lentiscus* L. resin produced comparable inhibition zones against all tested bacterial strains despite being applied at a fixed volume of 20 µL. In contrast, gentamicin—used as a positive control—was applied at a concentration of 1 mg/mL (Table 2). In the microdilution assay, the essential oil exhibited notable antimicrobial activity, with the most significant effects observed against *P. aeruginosa* (MIC = 0.12% *v*/*v*) among Gram-negative bacteria and *M. luteus* (MIC = 0.06% *v*/*v*) among Gram-positive bacteria.

In the antifungal assay, *P. lentiscus* L. resin essential oil was tested at a fixed volume of 20 µL, while cycloheximide, used as a positive control, was applied at a concentration of 1 mg/mL Despite this difference in applied concentrations, the essential oil produced inhibition zones comparable to those of cycloheximide against the yeast *C. glabrata* and the mold *A. niger* (Table 3).

In the microdilution assay, the essential oil demonstrated moderate antifungal activity, with a MIC value of 2% *v*/*v* for both fungal strains.

### 3.5. Molecular Docking

Among the bioactive compounds analyzed, caryophyllene and its oxide exhibited the lowest binding energy values across all selected target proteins involved in antibacterial, antifungal, and antioxidant activities (Table 4). These results suggest that both compounds have a strong binding affinity to the target proteins, potentially contributing to the pronounced bioactivity observed in the essential oil.

Additionally, D-limonene showed the highest binding affinity to the fungal cytochrome P450 protein (PDB ID: 5FSA), indicating its possible role in the antifungal activity of the resin essential oil.

**Figure 3:**
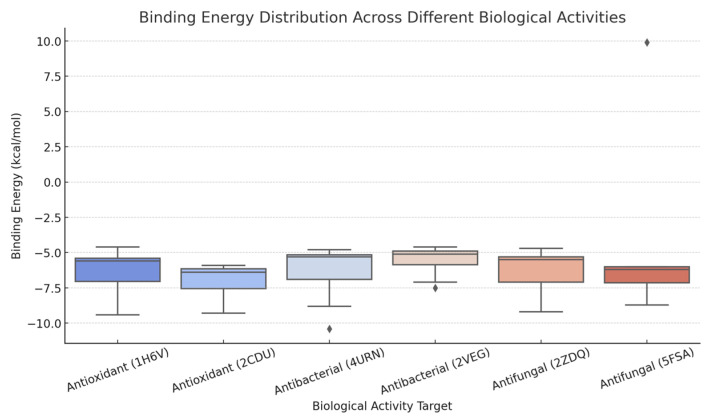
Binding energy distribution of major constituents of *Pistacia lentiscus* L. resin essential oil across proteins associated with antibacterial (4URN), antioxidant (1H6V), and antifungal (5FSA) activities. Box plot visualizations were generated using descriptive statistical data to illustrate variations in binding affinities (∆G, kcal/mol) among the compounds.

Using descriptive statistics data, box plot visualizations were made in Figure 3 to visualize the differences in binding energies. The analysis reveals that:

- Antibacterial activity (4URN) displays the widest distribution of binding energies, indicating significant variability in compound-protein interactions.
- Antioxidant activity (1H6V) is associated with a relatively low mean binding energy (−6.22 kcal/mol), with minimal standard deviation, suggesting more consistent binding across compounds.
- Antifungal activity (5FSA) exhibits the highest standard deviation (4.38), reflecting notable differences in binding efficiency among tested compounds.

## 4. Discussion

The essential oil of *P. lentiscus* L. resin, which is widely distributed in eastern Morocco, was successfully obtained through conventional hydrodistillation. The yield (1.4%) was approximately half of that reported for Chios mastic gum essential oil [2], yet it exceeded the minimum requirement of 10 mL/kg of anhydrous drug as specified by the European Pharmacopoeia [24].

Chemically, the Moroccan resin oil was primarily composed of α-pinene, β-pinene, and D-limonene, which together represented 55% of the total constituents. This is consistent with previous findings from our earlier study on the stem essential oil of *P. lentiscus* L., where α-pinene also dominated the composition, reinforcing its role as a key compound synthesized in response to bark injury [21]. While α-pinene is also the principal component of Chios mastic gum essential oil, the overall chemical profiles differ between the two resins. These compositional differences are likely due to environmental factors such as climate, soil type, and altitude [25].

Most studies have focused on the essential oil composition of *P. lentiscus* L. leaves and fruits, with limited attention given to resin variability across different environments. However, findings from foliar and fruit studies suggest that secondary metabolite profiles are highly influenced by environmental conditions. For instance, *P. lentiscus* L. leaves collected from littoral and mountain zones in Algeria showed distinct chemical profiles, attributed to climatic differences [15]. Similarly, research in Spain demonstrated that soil parameters and bioclimatic factors significantly affect essential oil composition in *P. lentiscus* L. leaves [16]. These insights emphasize the need for further research specifically targeting the resin to clarify how environmental variables shape its chemistry and bioactivity.

The extraction method used was also shown to significantly influence the chemical profile of the resin extracts. Among the techniques tested, US-SPME produced the highest proportion of monoterpenoids (91%), followed by solid-phase microextraction (SPME, 89%) and headspace (HS) extraction (71%). Interestingly, *cis*-ocimene—despite its relatively low volatility—was the dominant compound in both SPME and US-SPME extracts, likely due to the strong affinity of the CAR/PDMS fiber for linear monoterpenes [26]. In contrast, HS extraction, which is independent of fiber selectivity, captured a broader spectrum of volatiles, including higher levels of α-pinene and camphene [27].

Extraction parameters, particularly temperature and time, played a crucial role in determining both the yield and selectivity of volatile recovery. For SPME, an extraction temperature of 60 °C was selected based on literature demonstrating its effectiveness in enhancing the desorption of monoterpenes while minimizing thermal degradation. Šikuten et al. (2021) found that 60 °C offered optimal recovery without compromising the integrity of thermolabile compounds [28]. A 20 min extraction time was chosen as a balance between efficiency and analytical throughput, even though extended durations (up to 49 min) may yield slightly higher recoveries [28].

For US-SPME, extraction conditions were optimized to preserve the integrity of volatiles while enhancing efficiency. The temperature was maintained at 25 °C to prevent heat-induced degradation, a strategy supported by García Y. et al. [29] who reported that low temperatures preserved the volatile profile of acerola fruit [29]. The extraction time was reduced to 12 min, as ultrasonic agitation significantly enhances mass transfer and facilitates rapid volatile release without compromising compound stability. This finding is consistent with observations by Yang et al. (2021), who optimized SPME conditions for Laoxianghuang volatiles [30].

US-SPME specifically enhanced the extraction of certain compounds such as *m*-cymene, though it resulted in slightly lower levels of *cis*-ocimene and α-pinene compared to SPME. This suggests that ultrasound selectively improves the availability of some volatiles while potentially inhibiting the release of others, possibly due to structural interactions with the resin matrix [31].

In contrast, HS extraction at 80 °C facilitated the release of heavier monoterpenes and long-chain aldehydes such as *trans*-dodec-2-en-1-al and *E*-dec-2-enal. These compounds were absent in the SPME-based methods, indicating that their liberation or formation likely occurs through heat-induced oxidation or degradation processes [32].

Taken together, the compositional differences across the three extraction techniques can be attributed to variations in compound volatility, fiber selectivity, and extraction temperature. HS extraction produced a more diverse volatile profile, while SPME and US-SPME selectively concentrated compounds that are better retained by the fiber coating. The incorporation of ultrasound in US-SPME further modulated the extraction pattern, highlighting the importance of tailoring extraction strategies to the physicochemical properties of target compounds.

The essential oil demonstrated moderate antioxidant activity in both the DPPH and ABTS assays. Complementary molecular docking analysis revealed that caryophyllene and caryophyllene oxide exhibited the strongest binding affinities to key antioxidant-related target proteins (1H6V and 2CDU), suggesting their prominent role in the oil’s free radical scavenging activity. These findings are consistent with earlier reports highlighting the potent antioxidant potential of caryophyllene [33]. In addition, α-pinene, β-pinene, and D-limonene—also present in high concentrations in the oil—are well-documented for their antioxidant properties and likely contribute synergistically to the observed activity [34,35].

Although *P. lentiscus* L. resin essential oil has shown moderate radical-scavenging activity in direct assays such as DPPH and ABTS, previous studies suggest that its antioxidant potential may also involve indirect mechanisms. Xanthis et al. (2021) reported that essential oil rich in myrcene and α-pinene did not exhibit strong direct antioxidant activity in these assays; however, it significantly enhanced the expression of antioxidant response genes, indicating a cytoprotective effect mediated through upregulation of cellular defense pathways [36].

The essential oil also demonstrated promising antimicrobial activity, particularly against *Micrococcus luteus*, *Candida glabrata*, and *Aspergillus niger*. Docking simulations supported these findings, showing strong interactions between caryophyllene (and its oxide) and microbial target proteins. D-limonene exhibited a high affinity for 5FSA, a fungal cytochrome P450 enzyme, supporting its potential role in antifungal activity. However, the overall antimicrobial effects are likely the result of synergistic interactions among multiple constituents rather than the effect of any single compound [37].

This chemical and biological profile suggests potential applications for essential oil as a natural food preservative. Its effectiveness against spoilage-related pathogens—such as *P. aeruginosa*, *M. luteus*, and *A. niger*—points to its utility in prolonging the shelf life of fresh and processed food products [38,39,40]. In addition, the inhibition of *C. glabrata*, a common yeast in the human gut mycobiota and an opportunistic pathogen, suggests potential use in functional foods with protective effects [41].

Previous studies demonstrated synergy among terpenes in improving free radical scavenging activity. For instance, research has shown that γ-terpinene can regenerate chain-breaking antioxidants, while binary mixtures of monoterpenes, such as *p*-cymene with α-terpinene or α-phellandrene, have exhibited enhanced antioxidant properties [42]. Additionally, essential oil combinations have demonstrated increased antioxidant activity compared to individual components [43,44]. These findings support the potential of monoterpene synergy in *Pistacia lentiscus* L. essential oil, and further experimental validation is recommended.

It is known that combinations of phenolic compounds like thymol and carvacrol have demonstrated enhanced antimicrobial efficacy against *Escherichia coli* strains. Additionally, synergistic interactions between essential oils and antimicrobial peptides, such as Cecropin A, have been observed, suggesting broader applications in combating bacterial pathogens [45].

The potential for microbial adaptation or resistance following prolonged exposure to essential oils is an important consideration. It is known that the risk of resistance development to essential oils is lower than that observed with conventional antibiotics. Studies have shown that certain bacterial strains, such as *Serratia marcescens*, *Morganella morganii*, and *Proteus mirabilis*, exhibited some changes in their antibiotic resistance profiles after prolonged exposure to subinhibitory concentrations of oregano and cinnamon essential oils. However, these changes were not consistently observed across all tested strains, suggesting a limited potential for widespread resistance development. Additionally, research indicates that the multi-component nature of essential oils may reduce the likelihood of microbial adaptation, as bacteria would need to simultaneously develop resistance to multiple active compounds. This complexity makes spontaneous resistance less probable compared to single-target antibiotics. While the risk of adaptation remains a concern, current evidence suggests that essential oils maintain their antimicrobial efficacy over time, emphasizing the importance of continued research and responsible use to mitigate potential resistance [46,47].

According to Fratini et al. (2025), β-caryophyllene exhibits a synergistic antimicrobial effect in combination with antimicrobial peptides such as Cecropin A [45]. Furthermore, Ciesla et al. (2016) showed that synergistic interactions between terpenes (e.g., α-pinene and β-pinene) can enhance their antioxidant and antimicrobial properties [43].

These findings suggest that the antioxidant and antimicrobial activity of *Pistacia lentiscus* L. is the result of synergistic effects and not just the action of individual compounds.

## 5. Conclusions

This study investigated the composition, bioactivity, and extraction efficiency of *P. lentiscus* L. resin essential oil from eastern Morocco. The oil, obtained through hydrodistillation with a 1.4% yield, met European Pharmacopoeia standards despite yielding less than Chios mastic gum. α-Pinene, β-pinene, and D-limonene were the dominant components, collectively accounting for 55% of the total content, with compositional differences likely influenced by environmental factors.

The essential oil exhibited notable antioxidant and antimicrobial activity, highlighting its potential as a natural preservative or functional food ingredient. Molecular docking identified caryophyllene and its oxide as the most active compounds, showing the strongest interactions with antioxidant- and antimicrobial-related proteins. However, the overall bioactivity of the oil is likely attributed to the synergistic effects of its multiple constituents rather than the action of individual compounds alone.

A comparison of extraction methods highlighted the influence of volatility, fiber selectivity, and temperature on compound distribution. HS extraction favored highly volatile compounds, such as α-pinene and camphene, while SPME and US-SPME selectively enriched *cis*-ocimene and D-limonene due to fiber affinity. The incorporation of ultrasound in US-SPME further modified the volatile profile, increasing *m*-cymene while reducing α-pinene content.

This study has practical implications for the functional food industry, natural preservatives, and natural antioxidants. The obtained data will contribute to the optimization of extraction methods for bioactive compounds and support their potential use in the food, pharmaceutical, and cosmetic industries.

A systematic investigation of the chemical composition, biological activity, and extraction methods of *Pistacia lentiscus* L. resin essential oil from Eastern Morocco provides new insights to the scientific community and expands the applicability of this natural resource across various industries.

Future research should focus on identifying specific synergies among oil constituents and elucidating their mechanisms of action in real food systems and biological models. Additionally, optimizing extraction techniques could enhance yield and bioactive compound recovery, maximizing the oil’s functional potential for food preservation and therapeutic applications.

## Figures and Tables

**Table 1:** GC/MS analysis of *P. lentiscus* L. resin essential oil, obtained by hydrodistillation (HD) and resin extracts, obtained by head space (HS), solid-phase microextraction (SPME), and ultrasonic solid-phase microextraction (US-SPME). Percentages represent TIC% (Total Ion Chromatogram %).

Components	Formula	GC/MS Analysis				
		RT *	HD (%)	HS (%)	SPME (%)	US-SPME (%)
2-Propanone	C_3_H_6_O	0.02	n.d. *	n.d.	0.12 ^a^	0.06 ^a^
6,6-Dimethylhepta-2,4-diene	C_9_H_16_	0.64	n.d.	n.d.	0.42 ^a^	0.26 ^b^
Methanol, (1,4-dihydrophenyl)	C_7_H_10_O	2.06	n.d.	n.d.	n.d.	0.14
*cis*-Ocimene	C_10_H_16_	2.88	n.d.	n.d.	54.31 ^a^	46.28 ^b^
2-Carene	C_10_H_16_	3.02	n.d.	n.d.	0.15 ^a^	0.27 ^a^
Benzene, methyl	C_7_H_8_	3.18	n.d.	n.d.	2.07 ^b^	3.01 ^a^
But-3-ene	C_4_H_8_	3.53	n.d.	n.d.	0.34	n.d.
Heptanal	C_7_H_14_O	4.69	n.d.	0.75	n.d.	n.d.
Tricyclene	C_10_H_16_	5.05	0.96 ^b^	5.53 ^a^	0.76 ^c^	0.65 ^c^
3-Carene	C_10_H_16_	5.22	n.d.	7.05	n.d.	n.d.
α-Pinene	C_10_H_16_	5.41	25.98 ^a^	13.56 ^b^	n.d.	3.85 ^c^
Verbenene	C_10_H_14_	5.50	n.d.	n.d.	2.05 ^b^	3.07 ^a^
Camphene	C_10_H_16_	5.50	3.43	12.26	2.88	2.64
*trans*-Verbenol	C_10_H_16_O	5.56	2.46	n.d.	n.d.	n.d.
*p*-Mentha-1(7),8(10)-dien-9-ol	C_10_H_16_O	5.58	n.d.	6.70	n.d.	n.d.
Sabinene	C_10_H_16_	5.88	n.d.	2.99 ^a^	0.18 ^b^	n.d.
β-Pinene	C_10_H_16_	5.95	19.02 ^a^	9.68 ^b^	5.49 ^c^	0.16 ^d^
β-Myrcene	C_10_H_16_	6.11	3.42	n.d.	n.d.	n.d.
Octanal	C_8_H_16_O	6.33	n.d.	1.06	n.d.	n.d.
α-Phellandrene	C_10_H_16_	6.38	2.51	n.d.	n.d.	n.d.
4-Carene	C_10_H_16_	6.49	n.d.	1.43	n.d.	n.d.
*p*-Cymene	C_10_H_14_	6.74	3.67 ^a^	0.71 ^b^	n.d.	n.d.
D-Limonene	C_10_H_16_	6.89	9.96 ^a^	0.37 ^d^	6.23 ^b^	5.71 ^c^
*o*-Cymol	C_10_H_14_	6.97	0.25	n.d.	n.d.	n.d.
cis-β-Ocimene	C_10_H_16_	7.07	n.d.	0.20	n.d.	n.d.
Ocimene	C_10_H_16_	7.27	n.d.	0.37	n.d.	n.d.
*n*-Octanol	C_10_H_18_O	7.49	n.d.	0.34	n.d.	n.d.
*trans*-Limonene oxide	C_10_H_16_O	7.53	0.03	n.d.	n.d.	n.d.
(2*E*)-3-Pentyl-2,4-pentadien-1-ol	C_10_H_18_O	7.56	n.d.	0.26	n.d.	n.d.
*trans*-*p*-Mentha-2,8-dienol	C_10_H_16_O	7.78	0.56	n.d.	n.d.	n.d.
Thujol	C_10_H_18_O	7.82	0.55	n.d.	n.d.	n.d.
3-Tridecene	C_13_H_26_	8.01	n.d.	9.07	n.d.	n.d.
Verbenol	C_10_H_16_O	8.19	0.15 ^c^	2.3 ^a^	0.29 ^b^	2.10 ^a^
4-Heptenal, (*Z*)	C_7_H_12_O	8.20	n.d.	n.d.	n.d.	0.78
2,5-Dimethyl-1,5-hexadien-3-ol	C_9_H_14_O_3_	8.39	n.d.	n.d.	n.d.	0.25
α-Campholenal	C_10_H_16_O	8.45	3.03 ^a^	1.97 ^b^	1.11 ^c^	1.26 ^c^
Eucalyptol	C_10_H_18_O	8.45	n.d.	n.d.	0.37 ^a^	0.29 ^b^
*p*-Mentha-1,3,8-triene	C_10_H_14_	8.70	n.d.	n.d.	n.d.	0.67
*L*-Pinocarveol	C_10_H_16_O	8.73	2.62 ^a^	1.27 ^b^	n.d.	n.d.
*p*-Mentha-1,5,8-triene	C_10_H_14_	8.77	n.d.	n.d.	0.72	n.d.
*cis*-Verbenol	C_10_H_16_O	8.80	n.d.	1.74 ^a^	0.36 ^b^	0.15 ^c^
Cyclohexanol, 2-methyl-3-(1-methylethenyl)	C_12_H_18_O_2_	8.80	n.d.	n.d.	n.d.	0.15
2,6-Dimethyl-3,5,7-octatriene-2-ol, *E*, *E*	C_10_H_16_O	8.81	3.98	n.d.	n.d.	n.d.
*p*-Mentha-1,5-dien-8-ol	C_10_H_16_O	8.88	3.37 ^a^	n.d.	0.49 ^b^	0.27 ^c^
(*E*)-Non-2-enal	C_9_H_16_O	8.92	n.d.	0.63	n.d.	n.d.
Pinocarvone	C_10_H_14_O	9.07	n.d.	1.01 ^a^	0.51 ^c^	0.75 ^b^
Myrcenol	C_10_H_18_O	9.18	3.93	n.d.	n.d.	n.d.
Terpinen-4-ol	C_10_H_18_O	9.33	0.94 ^a^	n.d.	0.17 ^b^	0.12 ^b^
*p*-Cymen-8-ol	C_10_H_14_O	9.48	0.57	n.d.	n.d.	n.d.
*p*-Menth-1-en-8-ol	C_10_H_18_O	9.56	0.40	n.d.	n.d.	n.d.
Myrtenal	C_10_H_14_O	9.62	0.72 ^c^	1.01 ^a^	0.76 ^c^	0.88 ^b^
Myrtenol	C_10_H_14_O	9.72	0.95 ^a^	n.d.	0.49 ^b^	0.56 ^b^
Verbenone	C_10_H_14_O	9.83	1.60 ^c^	0.47 ^d^	2.42 ^b^	3.95 ^a^
Carveol	C_10_H_16_O	10.03	0.09	n.d.	n.d.	n.d..
Carvone	C_10_H_14_O	10.35	0.04	n.d.	n.d.	n.d.
(2*E*)-2-Decenal	C_10_H_18_O	10.51	n.d.	3.94	n.d.	n.d.
*m*-Cymene	C_11_H_16_	10.64	n.d.	n.d.	n.d.	10.15
Bornyl acetate	C_12_H_20_O_2_	10.93	0.98 ^a^	0.50 ^b^	n.d.	n.d.
2,4-Dodecadien-1-al	C_12_H_22_O	11.02	n.d.	0.82	n.d.	n.d.
2-Ethyl-4,5-dimethylphenol	C_10_H_14_O	11.32	1.00	n.d.	n.d.	n.d.
2,4-Decadienal	C_10_H_16_O	11.36	n.d.	1.37	n.d.	n.d.
Styrene	C_12_H_18_O_2_	11.46	n.d.	n.d.	0.14 ^b^	0.35 ^a^
*o*-Cymol	C_10_H_14_	11.68	n.d.	n.d.	0.74 ^b^	0.96 ^a^
δ-Elemene	C_15_H_24_	11.70	0.4 ^a^	n.d.	0.16 ^b^	0.17 ^b^
2-Undecenal	C_11_H_20_O	11.80	n.d.	0.63	n.d.	n.d.
α-Cubebene	C_15_H_24_	11.87	0.19 ^b^	0.64 ^a^	n.d.	n.d.
Ylangene	C_15_H_24_	11.97	0.25	n.d.	n.d.	n.d.
*trans*-Dodec-2-en-1-al	C_12_H_24_O	12.01	n.d.	5.16	n.d.	n.d.
Germacrene D-4-ol	C_15_H_26_O	12.46	0.54	n.d.	n.d.	n.d.
β-Elemene	C_15_H_24_	12.50	0.23	n.d.	n.d.	n.d.
D-Longifolene	C_15_H_24_	12.82	n.d.	0.61	n.d.	n.d.
6-Methyl-5-hepten-2-one	C_8_H_14_O	13.07	n.d.	n.d.	0.34 ^b^	0.45 ^a^
Caryophyllene	C_15_H_24_	12.97	0.59	n.d.	n.d.	n.d.
*cis*-α-Bisabolene	C_15_H_24_	13.45	0.03	n.d.	n.d.	n.d.
Copaene	C_15_H_24_	13.71	0.22	n.d.	n.d.	n.d.
Aromadendrene	C_15_H_24_	14.23	0.01	n.d.	n.d.	n.d.
δ-Cadinene	C_15_H_24_	14.31	0.01	n.d.	n.d.	n.d.
*Z*-Hexadec-8-ene	C_16_H_32_	14.83	n.d.	0.27	n.d.	n.d.
Caryophyllene oxide	C_15_H_24_O	15.22	0.35 ^a^	n.d.	n.d.	0.11 ^b^
Fencholenic aldehyde	C_10_H_16_O	15.86	n.d.	n.d.	0.20 ^a^	0.18 ^a^
(2*E*)-2-Tetradecen-1-ol	C_14_H_28_O	15.96	n.d.	0.35	n.d.	n.d.
(*E*)-14-Hexadecenal	C_16_H_30_O	16.03	n.d.	0.64	n.d.	n.d.
*p*-Cymenene	C_10_H_12_	16.20	n.d.	n.d.	n.d.	1.11
(*Z*)-Hexadec-7-enal	C_16_H_30_O	16.30	n.d.	0.66 ^b^	2.94 ^a^	n.d.
1,2-Cyclododecanediol	C_12_H_24_O_2_	18.37	n.d.	0.63 ^b^	0.75 ^a^	n.d.
13-Tetradecenal	C_12_H_26_O	18.57	n.d.	1.05	n.d.	n.d.
3-Pinanone	C_10_H_16_O	18.59	n.d.	n.d.	0.63 ^b^	0.83 ^a^
β-Burbonene	C_15_H_24_	18.71	n.d.	n.d.	0.34 ^b^	1.16 ^a^
2,4-Dimethyl-2,6-heptadienal	C_9_H_14_O	19.38	n.d.	n.d.	0.26 ^b^	0.30 ^a^
2,3,4,5-Tetramethyl-2-cyclopenten-1-one	C_9_H_14_O	20.44	n.d.	n.d.	n.d.	0.27
Bornyl acetate	C_12_H_20_O	20.71	n.d.	n.d.	0.41 ^a^	0.45 ^a^
2,3-Epoxypinane	C_10_H_16_O	20.85	n.d.	n.d.	0.52	n.d.
Elemol	C_15_H_26_O	20.95	n.d.	n.d.	n.d.	0.16
*trans*-β-Caryophyllene	C_15_H_24_	21.04	n.d.	n.d.	n.d.	0.28
*trans*-Verbenyl acetate	C_12_H_18_O_2_	21.41	n.d.	n.d.	0.88 ^a^	0.33 ^b^
Myrtenyl acetate	C_12_H_18_O_2_	22.48	n.d.	n.d.	0.33 ^b^	0.58 ^a^
*L*-*trans*-Pinocarveol	C_10_H_16_O	23.01	n.d.	n.d.	1.84 ^b^	1.97 ^a^
(*S*)-*cis*-Verbenol	C_10_H_16_O	23.11	n.d.	n.d.	2.33 ^a^	0.46 ^b^
Cyclohexene, 3-acetoxy-4-(1-hydroxy-1-methylethyl)-1-methyl-	C_12_H_20_O_3_	23.29	n.d.	n.d.	0.11	n.d.
*cis*-Carveol	C_10_H_16_O	28.04	n.d.	n.d.	0.36 ^a^	0.33 ^a^
Cumyl alcohol	C_10_H_14_O	28.36	n.d.	n.d.	n.d.	0.18
3-Cyclohexene-1-carboxylic acid, 3,4-dimethyl-, methyl ester	C_10_H_16_O	39.10	n.d.	n.d.	n.d.	0.13
Monoterpenes (%)			69.20 ^c^	54.15 ^d^	73.93 ^b^	75.78 ^a^
*O*-Containing monoterpenoids (%)			26.97 ^a^	16.97 ^b^	15.23 ^c^	15.64 ^c^
Total monoterpenoids (%)			96.17 ^a^	71.20 ^d^	89.16 ^c^	91.42 ^b^
Sesquiterpenes (%)			1.93 ^a^	1.25 ^c^	0 ^d^	1.61 ^b^
*O*-Containing sesquiterpenoids (%)			0.89 ^a^	0 ^c^	0 ^c^	0.27 ^b^
Total sesquiterpenoids (%)			2.82 ^a^	1.25 ^c^	0 ^d^	1.88 ^b^
Other compounds			1.00 ^d^	27.63 ^a^	6.47 ^b^	5.89 ^c^

* RT—retention time, min; n.d.—not detected; Values within a row followed by different superscript letters (a–d) are significantly different (*p* < 0.05) according to Duncan’s multiple range test. Values sharing the same letter are not significantly different.

**Table 2:** Antibacterial activity of *P. lentiscus* L. resin essential oil evaluated by agar diffusion and microdilution assays.

	*E. coli*	*P. aeruginosa*	*M. luteus*	*S. aureus*
Essential oil (20 µL)	10.33 ± 0.30 *	10.26 ± 0.25	12.63 ± 0.41	11.90 ± 0.45
Gentamicin (1 mg/mL)	25.20 ± 0.40 *	25.83 ± 0.41	26.76 ± 0.45	27.16 ± 0.41
MIC (% *v*/*v*)	0.5	0.125	0.062	0.75
MBC (% *v*/*v*)	≥8	≥8	≥8	≥8

* Diameter of the inhibition zone, mm.

**Table 3:** Antifungal activity of *P. lentiscus* L. resin essential oil evaluated by agar diffusion and microdilution assays.

	*R. aureus*	*C. glabrata*	*A. niger*	*G. candidum*
Essential oil (20 µL)	11.50 ± 0.26 *	18.8 ± 0.79	19.56 ± 0.80	13.30 ± 0.45
Cycloheximide (1 mg/mL)	21.10 ± 0.45 *	21.5 ± 0.20	23.70 ± 0.36	22.76 ± 0.40
MIC (% *v*/*v*)	≥8	2	2	8
MBC (% *v*/*v*)	≥8	≥8	≥8	≥8

* Diameter of the inhibition zone, mm.

**Table 4:** Binding energy (∆G, kcal/mol) of the main constituents of *P. lentiscus* L. resin essential oil against selected target proteins associated with antioxidant, antibacterial, and antifungal activities. Lower ∆G values indicate stronger predicted binding affinity.

	Antioxidant Activity		Antibacterial Activity		Antifungal Activity	
	1H6V	2CDU	4URN	2VEG	2ZDQ	5FSA
Compounds	Binding Free Energy ∆G (kcal/mol)					
Ligand Natif	−8.0	−7.7	−10.4	−6.9	−8.1	9.9
α-Pinene	−5.5	−6.4	−5.0	−5.1	−5.4	−6.1
β-Pinene	−5.2	−6.4	−5.2	−5.3	−5.3	−6.0
D-Limonene	−5.6	−6.2	−5.3	−4.9	−5.3	**−7.1**
Myrcenol	−5.3	−5.9	−5.1	−4.7	−4.7	−6.0
*p*-Cymene	−5.6	−6.4	−5.3	−5.0	−5.6	−7.2
Camphene	−5.5	−6.4	−4.9	−4.9	−5.1	−6.2
β-Myrcene	−4.6	−6.1	−4.8	−4.6	−4.7	−6.6
α-Campholenal	−5.3	−6.1	−5.3	−4.8	−5.5	−6.0
*L*-Pinocarveol	−5.6	−6.0	−5.4	−5.2	−5.5	−6.0
Verbenone	−5.6	−6.7	−5.3	−4.9	−5.7	−6.1
Caryophyllene	**−6.9**	−7.6 *	**−7.1**	**−5.8**	**−6.9**	**−7.2**
Caryophyllene oxide	**−7.2**	**−7.5**	**−6.7**	**−5.9**	**−7.3**	**−6.9**
Gentamicin	−9.4	−9.3	−8.8	−7.1	−9.2	−8.7
Cycloheximide	−8.0	−8.3	−8.0	−7.5	−7.8	−8.3

* Values in bold refer to the lowest binding energy, i.e., highest affinity to target proteins.

## Data Availability

The original contributions presented in this study are included in the article. Further inquiries can be directed to the corresponding authors.

## Abbreviations

The following abbreviations are used in this manuscript:

HD	Headspace
SPME	Solid-phase microextraction
US-SPME	Ultrasonic solid-phase microextraction
GC-MS	Chromatography–mass spectrometry
PTFE	Polytetrafluoroethylene
PDB	Potato dextrose broth
PDB	Protein Data Bank

## References

[1] Soulaidopoulos S., Tsiogka A., Chrysohoou C., Lazarou E., Aznaouridis K., Doundoulakis I., Tyrovola D., Tousoulis D., Tsioufis K., Vlachopoulos C. (2022). Overview of Chios Mastic Gum (*Pistacia lentiscus*) Effects on Human Health. Nutrients.

[2] Paraskevopoulou A., Kiosseoglou V., Kristbergsson K., Ötles S. (2016). Chios Mastic Gum and Its Food Applications. Functional Properties of Traditional Foods; Integrating Food Science and Engineering Knowledge into the Food Chain.

[3] Pachi V.K., Mikropoulou E.V., Gkiouvetidis P., Siafakas K., Argyropoulou A., Angelis A., Mitakou S., Halabalaki M. (2020). Traditional Uses, Phytochemistry and Pharmacology of Chios Mastic Gum (*Pistacia lentiscus* var. *Chia*, Anacardiaceae): A Review. J. Ethnopharmacol..

[4] Gortzi O., Rovoli M., Katsoulis K., Graikou K., Karagkini D.-A., Stagos D., Kouretas D., Tsaknis J., Chinou I. (2022). Study of Stability, Cytotoxic and Antimicrobial Activity of Chios Mastic Gum Fractions (Neutral, Acidic) After Encapsulation in Liposomes. Foods.

[5] Ganos C.G., Aligiannis N., Chinou I., Mérillon J.M., Riviere C., Lefèvre G. (2023). Selected Traditional Beverages from Greece (North Aegean Region and Crete): History, Comprehensive Evaluation, and Future Perspectives. Natural Products in Beverages. Reference Series in Phytochemistry.

[6] Hadini A., Azdimousa A., Khoulati A., El Bekkaye K., Saalaoui E. (2022). Valorization of Moroccan *Pistacia lentiscus* L. Leaves: Phytochemical and In Vitro Antioxidant Activity Evaluation Compared to Different Altitudes. Sci. World J..

[7] Koehler J. (2012). Morocco: A Culinary Journey with Recipes from the Spice-Scented Markets of Marrakech to the Date-Filled Oasis of Zagora.

[8] Mohagheghzadeh A., Faridi P., Ghasemi Y. (2010). Analysis of Mount Atlas Mastic Smoke: A Potential Food Preservative. Fitoterapia.

[9] Al-Zaben M., Zaban M.A., Naghmouchi S., Nasser Alsaloom A., Al-Sugiran N., Al-rokban A. (2023). Comparison of Phytochemical Composition, Antibacterial, and Antifungal Activities of Extracts from Three Organs of *Pistacia lentiscus* from Saudi Arabia. Molecules.

[10] Jaradat N., Al-Maharik N., Hawash M., Zaid A.N., Eid A.M., Hudaib M., Bustanji Y., Zihlif M., Issa R., Al-Qirim T. (2022). Essential Oil Composition, Antimicrobial, Cytotoxic, and Cyclooxygenase Inhibitory Areas of Activity of *Pistacia lentiscus* from Palestine. Arab. J. Sci. Eng..

[11] Sehaki C., Jullian N., Ayati F., Fernane F., Gontier E. (2023). A Review of *Pistacia lentiscus* Polyphenols: Chemical Diversity and Pharmacological Activities. Plants.

[12] Floris S., Di Petrillo A., Pintus F., Delogu G.L. (2024). Pistacia lentiscus: Phytochemistry and Antidiabetic Properties. Nutrients.

[13] Vitalini S., Iriti M., Garzoli S. (2022). GC-MS and SPME-GC/MS Analysis and Bioactive Potential Evaluation of Essential Oils from Two *Viola* Species Belonging to the *V. calcarata* Complex. Separations.

[14] Kowalczyk A., Kuś P., Marijanović Z., Tuberoso C.I.G., Fecka I., Jerković I. (2022). Headspace Solid-Phase Micro-Extraction Versus Hydrodistillation of Volatile Compounds from Leaves of Cultivated *Mentha* Taxa: Markers of Safe Chemotypes. Molecules.

[15] Sehaki C., Jullian N., Choque E., Dauwe R., Fontaine J.X., Molinie R., Ayati F., Fernane F., Gontier E. (2022). Profiling of Essential Oils from the Leaves of Pistacia lentiscus Collected in the Algerian Region of Tizi-Ouzou: Evidence of Chemical Variations Associated with Climatic Contrasts between Littoral and Mountain Samples. Molecules.

[16] Llinares J., Llorens-Molina J.-A., Mulet J., Vacas S. (2021). Soil Parameters and Bioclimatic Characteristics Affecting Essential Oil Composition of Leaves of *Pistacia lentiscus* L. from València (Spain). Span. J. Soil Sci..

[17] Zhao J., Quinto M., Zakia F., Li D. (2023). Microextraction of Essential Oils: A Review. J. Chromatogr. A.

[18] Bouakline H., Brahmi M., Ziani I., Rhizlan A., Idrissi Yahyaoui M., Angioni A., Talhaoui A., Bnouham M., Asehraou A., Tahani A. (2024). Influence of Air-Drying Temperature on Yield, Volatilome Content, Antioxidant, Antidiabetic and Antimicrobial Activities of *Pistacia lentiscus* Leaf Oil: Experimental and Modeling Aspects. Food Biosci..

[19] Pachi V.K., Mikropoulou E.V., Dimou S., Dionysopoulou M., Argyropoulou A., Diallinas G., Halabalaki M. (2021). Chemical Profiling of *Pistacia lentiscus* var. *Chia Resin* and Essential Oil: Ageing Markers and Antimicrobial Activity. Processes.

[20] John Wiley & Sons, National Institute of Standards and Technology (2011). Wiley9/NIST11 (W9N11) Mass Spectral Library.

[21] Beraich A., El Farissi H., Belbachir Y., Cacciola F., Yahyaoui M.I., Choukoud A., Talhaoui A. (2024). Traditional and Modern Extraction Methods for *Pistacia lentiscus* Essential Oil. Sustain. Chem. Pharm..

[22] Ahmed D., Khan M.M., Saeed R. (2015). Comparative Analysis of Phenolics, Flavonoids, and Antioxidant and Antibacterial Potential of Methanolic, Hexanic and Aqueous Extracts from
*Adiantum caudatum* Leaves. Antioxidants.

[23] Remmal A., Bouchikhi T., Rhayour K., Ettayebi M., Tantaoui-Elaraki A. (1993). Improved Method for the Determination of Antimicrobial Activity of Essential Oils in Agar Medium. J. Essent. Oil Res..

[24] Council of Europe (2017). Herbal Drugs and Herbal Drug Preparations. European Pharmacopoeia 9.0.

[25] Ottria R., Xynomilakis O., Casati S., Abbiati E., Maconi G., Ciuffreda P. (2023). Chios Mastic Gum: Chemical Profile and Pharmacological Properties in Inflammatory Bowel Disease: From the Past to the Future. Int. J. Mol. Sci..

[26] Yassaa N., Custer T., Song W., Pech F., Kesselmeier J., Williams J. (2010). Quantitative and Enantioselective Analysis of Monoterpenes From Plant Chambers and in Ambient Air Using SPME. Atmos. Meas. Tech..

[27] Song N.E., Lee J.Y., Lee Y.Y., Park J.-D., Jang H.W. (2019). Comparison of Headspace–SPME and SPME-Arrow–GC–MS Methods for the Determination of Volatile Compounds in Korean Salt–Fermented Fish Sauce. Appl. Biol. Chem..

[28] Šikuten I., Štambuk P., Karoglan Kontić J., Maletić E., Tomaz I., Preiner D. (2021). Optimization of SPME-Arrow-GC/MS Method for Determination of Free and Bound Volatile Organic Compounds from Grape Skins. Molecules.

[29] García Y., Rufini J., Campos M., Guedes M.S., Augusti R., Melo J. (2018). SPME Fiber Evaluation for Volatile Organic Compounds Extraction from Acerola. J. Braz. Chem. Soc..

[30] Yang D.S., Lei Z., Bedair M., Sumner L.W. (2021). An Optimized SPME-GC-MS Method for Volatile Metabolite Profiling of Different Alfalfa (*Medicago sativa* L.) Tissues. Molecules.

[31] Chalvantzi I., Nisiotou A., Banilas G., Mallouchos A. (2023). Development of an Ultrasound-Assisted Emulsification Microextraction Method for the Determination of Volatile Compounds in Wines. Separations.

[32] Zhang Q., Qin W., Lin D., Shen Q., Saleh A.S. (2015). The Changes in the Volatile Aldehydes Formed During the Deep-Fat Frying Process. J. Food Sci. Technol..

[33] Tsigoriyna L., Sango C., Batovska D. (2024). An Update on Microbial Biosynthesis of β-Caryophyllene, a Sesquiterpene With Multi-Pharmacological Properties. Fermentation.

[34] Salehi B., Upadhyay S., Erdogan Orhan I., Kumar Jugran A., Jayaweera S.L.D., Dias D.A., Sharopov F., Taheri Y., Martins N., Baghalpour N. (2019). Therapeutic Potential of α- and β-Pinene: A Miracle Gift of Nature. Biomolecules.

[35] Ewais O., Abdel-Tawab H., El-Fayoumi H., Aboelhadid S.M., Al-Quraishy S., Falkowski P., Abdel-Baki A.S. (2024). Antioxidant Properties of D-Limonene and Its Nanoemulsion Form Enhance Its Anticoccidial Efficiency in Experimentally Infected Broilers With *Eimeria tenella*: An In Vitro and In Vivo Study. Vet. Res. Commun..

[36] Xanthis V., Fitsiou E., Voulgaridou G.P., Bogadakis A., Chlichlia K., Galanis A., Pappa A. (2021). Antioxidant and Cytoprotective Potential of the Essential Oil *Pistacia lentiscus* var. chia and Its Major Components Myrcene and α-Pinene. Antioxidants.

[37] Chouhan S., Sharma K., Guleria S. (2017). Antimicrobial Activity of Some Essential Oils—Present Status and Future Perspectives. Medicines.

[38] Liao S., Gong G., Wang X., Tian L. (2022). Membrane Damage Mechanism of Protocatechualdehyde Against *Micrococcus luteus* and Its Effect on Pork Quality Characteristics. Sci. Rep..

[39] Liang J., Huang T.Y., Li X., Gao Y. (2023). Germicidal Effect of Intense Pulsed Light on *Pseudomonas aeruginosa* in Food Processing. Front. Microbiol..

[40] Navale V., Vamkudoth K.R., Ajmera S., Dhuri V. (2021). *Aspergillus*-Derived Mycotoxins in Food and the Environment: Prevalence, Detection, and Toxicity. Toxicol. Rep..

[41] Al-Yasiri M., Normand A.C., L’Ollivier C., Lachaud L., Bourgeois N., Rebaudet S., Piarroux R., Mauffrey J.F., Ranque S. (2016). Opportunistic Fungal Pathogen *Candida glabrata* Circulates Between Humans and Yellow-Legged Gulls. Sci. Rep..

[42] Guo Y., Baschieri A., Amorati R., Valgimigli L. (2021). Synergic antioxidant activity of γ-terpinene with phenols and polyphenols enabled by hydroperoxyl radicals. Food Chem..

[43] Ciesla Ł., Wojtunik-Kulesza K., Oniszczuk A., Waksmundzka-Hajnos M. (2016). Antioxidant synergism and antagonism between selected monoterpenes using the 2,2-diphenyl-1-picrylhydrazyl method. Flavour Fragr. J..

[44] Benyoucef F., Dib M.E.A., Arrar Z., Costa J., Muselli A. (2018). Synergistic Antioxidant Activity and Chemical Composition of Essential Oils from *Thymus fontanesii*, *Artemisia herba-alba* and *Rosmarinus officinalis*. J. Appl. Biotechnol. Rep..

[45] Fratini F., Pecorini C., Resci I., Copelotti E., Nocera F.P., Najar B., Mancini S. (2025). Evaluation of the Synergistic Antimicrobial Activity of Essential Oils and Cecropin a Natural Peptide on Gram-Negative Bacteria. Animals.

[46] Becerril R., Nerín C., Gómez-Lus R. (2012). Evaluation of Bacterial Resistance to Essential Oils and Antibiotics after Exposure to Oregano and Cinnamon Essential Oils. Foodborne Pathog. Dis..

[47] Yap P.S., Yiap B.C., Ping H.C., Lim S.H. (2014). Essential Oils, a New Horizon in Combating Bacterial Antibiotic Resistance. Open Microbiol. J..

